# From Objects to Landmarks: The Function of Visual Location Information in Spatial Navigation

**DOI:** 10.3389/fpsyg.2012.00304

**Published:** 2012-08-27

**Authors:** Edgar Chan, Oliver Baumann, Mark A. Bellgrove, Jason B. Mattingley

**Affiliations:** ^1^Queensland Brain Institute, The University of QueenslandSt Lucia, QLD, Australia; ^2^School of Psychology, The University of QueenslandSt Lucia, QLD, Australia; ^3^School of Psychology and Psychiatry, Monash UniversityClayton, VIC, Australia

**Keywords:** landmarks, navigation, spatial memory, parahippocampal gyrus, topographical disorientation, hippocampus, striatum, retrosplenial cortex

## Abstract

Landmarks play an important role in guiding navigational behavior. A host of studies in the last 15 years has demonstrated that environmental objects can act as landmarks for navigation in different ways. In this review, we propose a parsimonious four-part taxonomy for conceptualizing object location information during navigation. We begin by outlining object properties that appear to be important for a landmark to attain salience. We then systematically examine the different functions of objects as navigational landmarks based on previous behavioral and neuroanatomical findings in rodents and humans. Evidence is presented showing that single environmental objects can function as navigational beacons, or act as associative or orientation cues. In addition, we argue that extended surfaces or boundaries can act as landmarks by providing a frame of reference for encoding spatial information. The present review provides a concise taxonomy of the use of visual objects as landmarks in navigation and should serve as a useful reference for future research into landmark-based spatial navigation.

## Introduction

Spatial navigation refers to the act of traversing the environment in a purposeful manner. At its simplest, spatial navigation involves moving from one location in space to another. Successful navigation is a fundamental cognitive function that is crucial for survival (e.g., foraging). Impairments in navigation can have widespread detrimental effects in everyday life (Aguirre and D’Esposito, 1999). Over millions of years animals have developed a variety of strategies for successful navigation, including taxon or locale strategies (O’Keefe and Nadel, 1978), place versus response learning strategies (Tolman, 1948), as well as eidetic snapshot matching (Cartwright and Collett, 1982), amongst several others. In addition, different types of cues can be utilized from geocentric cues such as the earth’s magnetic field (e.g., Able, 1991; Wehner et al., 1996; Boles and Lohmann, 2003), idiothetic cues that incorporate vestibular and proprioceptive information (e.g., Collett and Collett, 2000), to visual cues such as optic flow (e.g., Srinivasan et al., 1996), beacon learning (e.g., Lee and Spelke, 2010), and map following (e.g., Ruddle et al., 1999). In humans, everyday navigation involves a combination of one or more of these methods, but the use of visual information appears to be predominant (Foo et al., 2005). In this review, we use evidence from the relevant rodent and human literature to examine the different ways in which visual stimuli act as landmarks to guide navigation-related behavior.

## Approaches to Understanding Landmark Processing

**Figure 1:**
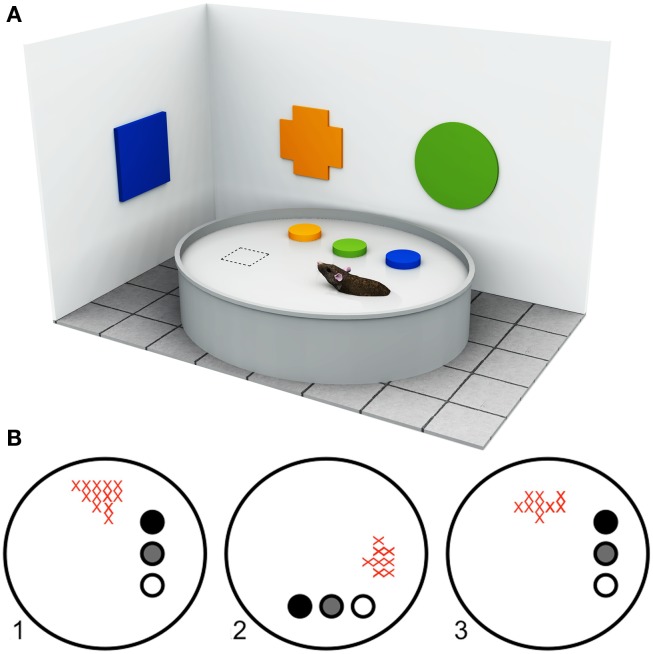
**Approaches to understanding landmark processing in rodents**. **(A)** Example of a typical Morris Water Maze setup. The Morris Water Maze paradigm (Morris, 1981), originally developed for rodents, requires the animal to locate a hidden platform (dashed square) that is submerged below the surface of a large circular arena filled with opaque water, using available room cues (in this illustration, the colored geometric shapes). The start position is varied between trials so that animals cannot use proprioceptive or egocentric strategies, but must learn to use the configural relationships between objects within the room to locate the platform. By manipulating the available visual information, or by lesioning specific neural structures, it is possible to determine the types of cues and brain regions that are important for solving the task. **(B)** Firing rate maps of a hippocampal place cell adapted from Cressant et al. (1997). “Place cells” are spatially selective cells, found in the rodent hippocampus, that fire maximally when the rodent is within a well-defined region of the environment (i.e., within the cell’s “place field”) but not at any other location, independent of head and body direction. Place cell activity is thought to reflect a rodent’s internal spatial representation of the environment. As such, the navigational relevance of different types of environmental object can be inferred from the manner in which they influence place cell activity. The firing rates of a rodent hippocampal place cell within a circular arena are shown in the example. The filled circles represent three prominent objects within the arena. As the spatial locations of objects are rotated between trials (1–3), the place field of the place cell shifts accordingly (red crosses). In this example, the rodent’s internal spatial representation is anchored by the three objects.

The term “landmark” has been used to describe many different types of visual information within a variety of contexts. Colloquially, the term is normally used to refer to well-known or visually salient buildings or monuments, such as the Eiffel Tower in Paris or the Opera House in Sydney. Over the last decades, research in rodents and humans has explored the role of visual landmarks in spatial navigation and memory using a variety of approaches. In rodents, the development of such tasks as the Morris Water Maze paradigm and the discovery of cells in the hippocampus (“place cells”) that appear to code for an animal’s spatial location (O’Keefe and Dostrovsky, 1971), have been important milestones in this undertaking (see Figure 1). Typically, however, the term “landmark” has been used in a rather idiosyncratic way. In the scientific literature on navigation and spatial behavior, the idea of a landmark has been applied very broadly to encompass any visual stimulus within an environment that could potentially influence navigation (e.g., Aguirre and D’Esposito, 1999; Caduff and Timpf, 2008; Tommasi et al., 2012). To date, there has been no attempt to draw together the navigation literature as a whole to examine whether landmarks can be characterized as subserving essentially similar functions, or whether it is possible to divide them into different functional subcategories. In this review, we aim to address this question by providing a parsimonious account of the roles of landmarks in navigation. In doing so, we hope to provide a reference for research on navigation, both in the laboratory and in clinical contexts.

We begin by examining the physical properties that appear to be important for imbuing ordinary visual objects with “landmark” status. We then examine the different ways in which environmental objects influence navigation-related behavior, and discuss the neural processes thought to be involved. We argue that the categorization of objects as landmarks should be based on the function of the object within a specific navigational context.

## Basic Physical and Psychological Properties of Objects as Landmarks

Given the wealth of available visual information in the world, how do objects in the environment attain their salience as navigational landmarks? Are there particular properties of visual objects that make them more or less likely to attain status as a landmark? With respect to physical appearance, the more unique an object is within an environment, and the more informative its features are, the more likely it is that it will be used as a landmark (Stankiewicz and Kalia, 2007). For example, a distinctive church tower may act as a salient landmark for determining one’s heading direction. By contrast, a tree or a road sign might be less informative if there are many similar objects within the same environment. Within a built-up environment, buildings that are more salient – large, uniquely shaped or colored, free standing, and so on – are better remembered in the course of way-finding (Evans et al., 1982; Miller and Carlson, 2011).

The stability of objects in the environment can also influence their salience as landmarks. In order for objects to provide reliable navigational information, they need to be perceived as having a stable spatial position. In rodents (Biegler and Morris, 1996), as well as humans (Burgess et al., 2004), the presence of stable objects in the environment can significantly improve navigational performance. The importance of landmark stability is further corroborated by studies in rodents showing that an object’s influence on hippocampal place cell activity is lost when the animal observes that the spatial location of the object has moved over time (Jeffery, 1998; Jeffery and O’Keefe, 1999). In these instances, animals learn from experience that the environmental objects are not reliable sources for determining their spatial location, and instead use idiothetic cues (i.e., internal movement cues) for orientation (Knierim et al., 1995). There is also a recent line of human fMRI evidence, which underlies the importance of landmark stability. Mullally and Maguire (2011) showed that the intrinsic stability of an object modulates neural activity in the medial temporal lobe. Participants viewed or imagined individual objects of varying sizes and categories. Objects that were rated by participants as larger and less “portable” elicited an increase in activity within the parahippocampal gyrus. Given the involvement of the parahippocampal gyrus in scene perception and landmark processing tasks (Epstein et al., 1999), it may be that more stable objects automatically evoke landmark-based neural processes. In line with this, it has also been shown that making spatial judgments with reference to stable environmental objects (e.g., a large building or a fountain) compared with unstable objects (e.g., a ball) elicit greater activity in navigationally relevant medial parietal and temporal brain regions, including the hippocampus (Committeri et al., 2004; Galati et al., 2010).

**Figure 2:**
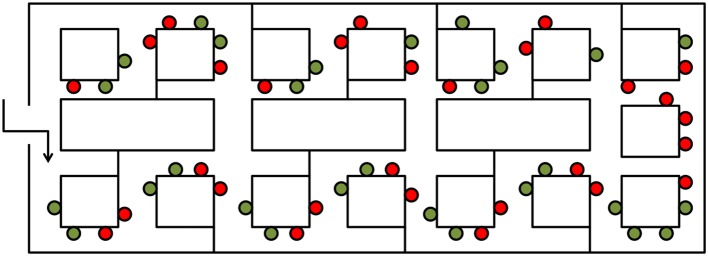
**The influence of spatial location on object processing adapted from Janzen and van Turennout (2004)**. An aerial schematic of the virtual museum used by Janzen and van Turennout (2004). Red dots indicate locations of decision point objects, and green dots indicate locations of non-decision point objects.

In addition to physical appearance and stability, the specific location of objects within an environment can also determine whether they attain salience as landmarks. Within a built-up environment, objects that occupy locations at which a navigational decision has to be made (e.g., a T-intersection) are more important for verbal route descriptions than objects at locations at which a navigational decision does not have to be made (e.g., an L-intersection or along a straight path; Cohen and Schuepfer, 1980; Blades and Medlicott, 1992). In line with this, it has been shown that objects at decision points are better remembered than those at non-decision points (Janzen, 2006; Kessels et al., 2011). In a seminal fMRI study, Janzen and van Turennout (2004) found that activity in the human parahippocampal gyrus was enhanced for objects at decision points compared with objects at non-decision points. Participants were passively guided through a virtual museum containing two types of objects (“toys” and “non-toys”) that were placed along the wall of the museum. Some of the objects were placed next to an intersection (a decision point), whereas others were placed along a straight path or at an L-shaped corner (non-decision points; see Figure 2). Participants were told they were training to be museum guides, and their task was to remember all the objects and to pay particular attention to the toys as opposed to the non-toy objects. After training, participants completed an old-new object recognition task for all objects in the museum while undergoing fMRI. Reaction times were faster for objects located at decision points than for those at non-decision points, and for toys compared with non-toys. Functional MRI data showed increased activity in the left and right parahippocampal gyri for objects at decision points relative to those at non-decision points. Notably, this effect appeared to be independent of participants’ attention to objects, as there was no significant interaction between object type and object location. That is, the effect was not different for toys and non-toys, despite the fact that participants were told to pay greater attention to toy objects during encoding. Furthermore, decision point related increases in parahippocampal activity were observed for remembered as well as for forgotten objects, suggesting that the coding of navigational relevance is an automatic process that does not require explicit memory. Follow-up studies by Janzen and colleagues have replicated and extended these important findings (Janzen and Weststeijn, 2007; Janzen et al., 2007, 2008).

More recently, Schinazi and Epstein (2010) examined whether such decision point effects in navigation could be replicated within a real-world setting. Students from the University of Pennsylvania were guided around their campus according to a pre-defined route and told to remember the buildings along the path. Consistent with previous studies, buildings at decision points were more readily recognized than buildings at non-decision points. Subsequent fMRI revealed increased parahippocampal activity for pictures of buildings that had appeared at decision points than for those at non-decision points, but only for buildings that occupied less familiar parts of the campus. The latter finding suggests that parahippocampal activity might reflect positional information during the early stages of route learning. Once an internal representation of the environment is established, parahippocampal activity no longer distinguishes between decision and non-decision points.

Findings from neuropsychological case studies provide further evidence consistent with the view that the parahippocampal gyrus might be important for the encoding and perception of navigationally relevant objects. Damage to the parahippocampal gyrus has been found to cause a form of topographical disorientation termed “landmark agnosia” in which the patient is selectively impaired in recognizing salient environmental landmarks, and thus exhibits significant way-finding deficits (Takahashi and Kawamura, 2002). Although these patients generally have preserved spatial representation abilities, with intact spatial learning (Whiteley and Warrington, 1978; Incisa della Rocchetta et al., 1996; McCarthy et al., 1996), they have difficulty finding their way around familiar and novel environments because they are unable to recognize salient landmarks during navigation, and fail to use them to provide directional information.

Taken together, the studies reviewed here suggest that physical and psychological object properties such as distinctiveness, stability, and position affect the likelihood that an object will be used as a landmark for navigation. A recent study by Miller and Carlson (2011) examined how some of these properties might interact in attaining landmark salience or navigational relevance within the same environment. Using a similar design as Janzen and van Turennout (2004), participants were required to attend to certain objects while guided through a virtual museum. However, objects in this study not only differed in spatial positioning (i.e., decision versus non-decision point) but also in perceptual salience (i.e., size and color). Findings showed that when these two cues are placed in conflict, objects with stronger perceptual features were later recognized faster irrespective of their spatial positioning. However, it was spatial positioning that determined whether objects were included in direction giving and map drawing. These findings suggest that the factors contributing to object salience for navigation are not strictly cumulative, but are most likely multi-dimensional and depend largely upon context and the goals of the individual (Caduff and Timpf, 2008). Even an intrinsically mundane object or building may be of special significance to an individual (e.g., an otherwise non-descript building might be a workplace or home) and may therefore attain strong navigational importance in some circumstances.

The most general and parsimonious definition of a landmark may be that this term refers to any object in the environment that is easily recognizable, as long as its primary function is that of a point of reference. To advance the understanding of landmark-based spatial navigation, however, it is advantageous to systematically differentiate landmark cues in terms of their specific role for navigation, rather than to use the term non-specifically for all objects that fall under the aforementioned general definition. At its simplest, visual objects can directly act as a beacon, marking an exact or nearby goal location. However, visual objects can also provide information about one’s current heading orientation. Furthermore, visual objects can be used as associative cues for eliciting navigationally relevant contextual information. Finally, visual objects can act as a landmark by providing a reference frame for the encoding of spatial information. In the following sections, we present a novel four-point taxonomy to define and summarize the different roles that visual objects play in spatial navigation.

## Objects as a Beacon for Navigation

At its simplest, a single object in the environment can serve as a navigational landmark by acting as a beacon. A beacon is an environmental object that indicates a nearby target location, or is itself a target location. For example, a lighthouse can act as a beacon to signal the location of land for those at sea. On the other hand, in a built-up environment, the spire of a church tower protruding from surrounding buildings can act as a beacon for finding the church. Beacon following is a form of visually guided navigation that is based upon a minimal allocentric reference frame, and involves monitoring the location of the self with respect to a single cue, irrespective of other environmental information.

Landmarks are often considered to fall in two broad categories: proximal and distal. Proximal landmarks are local environmental cues that provide accurate positional and route information, but are also perceived as being less reliable, given their often transient nature (Cheng and Spetch, 1998). Beacon cues share some of the characteristics of proximal cues in the sense that they provide accurate positional information. However, in contrast to proximal cues, beacon cues are, by definition, highly reliable predictors of a specific single goal location. Hence, an environment might contain several proximal landmarks, which might act as associative cues to provide route information (e.g., “right-turn at the traffic light”; see “Objects Used as Associative Cues” for more details), but a beacon cue is always exclusively associated with a single goal location. Furthermore, beacon cues provide accurate positional information for a single goal location, even if viewed from a considerable distance. However, the same environmental object might, under different task demands, serve other or additional navigational purposes. For example, the spire of a church can act as a beacon if the church is the goal, but as an orientation cue for other nearby goal locations.

The computational simplicity of beacon-based navigation, combined with its high reliability and positional precision, suggest that it is one of the most fundamental and primal navigational processes. This claim is supported by the finding that the use of objects as beacons for goal localization seems to emerge early in development in humans (Lehnung et al., 1998; MacDonald et al., 2004; Lee et al., 2006). Four-year old children, for example, are able to locate a target if it is hidden under a distinctive landmark, but not if the target is hidden under one of several identical landmarks and is thus distinguished only by its spatial location with respect to the surrounding environment (Lee et al., 2006).

Beacon-based navigation may continue to be the dominant strategy in adulthood, and in some cases may overshadow or block the encoding of more global spatial processes. In a series of experiments by Waller and Lippa (2007), adult participants learned a route along a virtual path using objects that either acted as beacons by providing a direct cue for the next goal location, or objects that acted as associative cues by providing a learned directional response for the next goal location. When objects served as beacons, route learning was more efficient than when they acted as associative cues. On the other hand, post-test assessment revealed that beacon-based route learning led to a less enduring representation of route information and a poorer representation of directional information. Furthermore, using a virtual Morris Water Maze (vMWM) in adult humans, it has been shown that the availability of beacon-like cues during learning is enough to block or overshadow the learning of other spatial information (e.g., Redhead et al., 1997; Hamilton and Sutherland, 1999; Roberts and Pearce, 1999; Hardt and Nadel, 2009).

Several lines of evidence, especially from rodent research, suggest that beacon-based navigation is supported by the dorsal striatum. For example, it had been shown that rats with damage to the dorsal striatum are unable to use beacon cues in spatial navigation tasks (Packard and McGaugh, 1992; McDonald and White, 1994). Furthermore, it had been found that metabolic activity associated with beacon-based navigation is associated with increased activity in the caudate nucleus, whereas increased activity in the hippocampus was found for allocentric strategies (Miranda et al., 2006). Tentative evidence to corroborate these findings comes from a recent fMRI study in humans, which observed striatal activity during navigation tasks where the goal location was indicated by a beacon cue (Baumann et al., 2010).

In summary, environmental objects can be landmarks for navigation by acting as a beacon. Beacon following appears to be one of the most basic forms of landmark-based navigation. It develops before other spatial strategies, and may be used in preference to other strategies due to its computational simplicity (Alyan and Jander, 1994; Alyan, 1996; Hardt and Nadel, 2009). Beacon-based navigation most probably depends on computational mechanisms controlled via the striatum, though further studies in humans will be needed to properly test this idea.

## Objects as Orientation or Directional Cues

Orientation in navigation generally refers to knowledge about one’s direction or heading with respect to the external world. Being oriented within an environment is important for successful navigation. For example, when lost in an unfamiliar place (e.g., in a shopping mall or a park), knowing one’s orientation can be useful for determining in which direction to head to find a way out. During navigation, a sense of direction is also essential for establishing an understanding about the spatial relationships between different locations in space, and can improve the stability of internal object location representations (Wang and Spelke, 2000). Depending on the organism, orientation or directional information will be controlled by a wide variety of cues. Thus, for example, it has been found that animals are able to use geocentric cues, such as the earth’s magnetic field, and the position of the sun and stars (Able, 1991; Wehner et al., 1996; Boles and Lohmann, 2003), internal idiothetic cues (Etienne and Jeffery, 2004), as well as visual objects that are within the immediate environment (Lew, 2011). However, in humans and rodents, when available and stable, it is principally visual cues that are used to control heading direction (e.g., Taube, 1998; Foo et al., 2005).

The importance of visual environmental objects in providing orientation or directional information is illustrated by neuropsychological case studies in which the ability to use these cues is disrupted. Patients with a condition known as “heading disorientation” have difficulty using visual environmental information to determine their current heading, despite possessing otherwise preserved visuo-spatial cognition and an ability to recognize the identity of landmarks (Barrash, 1998; Aguirre and D’Esposito, 1999; Greene et al., 2006; Tamura et al., 2007). These patients are generally found to have lesions within posterior-medial brain regions such as the retrosplenial cortex. The inability to determine orientation-related information from environmental objects leads to significant navigational impairments. For example, a 70-year-old woman who suffered a hemorrhage in the right medial parietal lobe had difficulty identifying viewpoints of particular buildings or landmarks, which led to significant navigational difficulties in the real-world (Suzuki et al., 1998). When shown photographs of her own house, she was unable to indicate the vantage point from which the photo was taken. Her inability to recognize the vantage point of buildings meant she could not establish a sense of direction for navigation, despite an intact ability to recognize familiar buildings and to complete abstract and memory-based map drawing and way-finding tasks. Another prominent series of case studies described three patients that apparently lost their sense of direction after sustaining right retrosplenial cortex lesions (Takahashi et al., 1997). They were found to have difficulty recalling the positional relationships between their current location and another destination within a space that could not be surveyed in its entirety at one time.

Further corroborating evidence for the existence of neural systems dedicated to the coding of heading direction comes from the rodent literature that suggests there are specialized neurons that code specifically for an animal’s orientation. These “head-direction” cells have been identified in a number of regions throughout the limbic system of the rodent brain including the anterodorsal thalamic nucleus, lateral mammillary nuclei, pre- and post-subiculum, and the retrosplenial cortex (Yoder et al., 2011). Head-direction cells exhibit firing activity that is tied to an animal’s heading during navigation, independent of its current location within the environment and its ongoing behavior (Taube, 1998). The preferred firing pattern of head-direction cells is mainly controlled by available visual cues. Rotation of salient objects around an arena causes similar rotations in the preferred firing direction of head-direction cells (e.g., Taube, 1998; Calton et al., 2008). Although idiothetic cues play some part in maintaining the firing properties of head-direction cells, as these cells continue to fire in darkness, such neurons quickly re-establish their firing pattern (within 80 ms) with respect to salient visual cues after illumination is restored (Zugaro et al., 2003). Furthermore, head-direction cells fire more consistently in response to environmental visual cues when visual and idiothetic information is artificially placed in conflict within an experimental arena (Goodridge and Taube, 1995; Taube and Burton, 1995; Zugaro et al., 2000). Also, it has recently been shown that head-direction cells are strongly influenced by discrete visual cues, whereas geometric cues only provide a weak influence for the computation of heading vectors (Knight et al., 2011).

**Figure 3:**
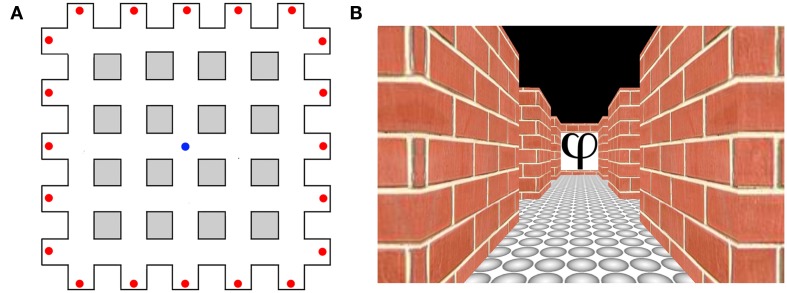
**Heading direction selectivity in humans adapted from Baumann and Mattingley (2010)**. **(A)** Aerial schematic of the virtual environment used in the study by Baumann and Mattingley (2010). Red dots represent the locations of the abstract symbols that acted as orientation landmarks. The blue dot represents the starting location of each learning trial. **(B)** An example of a single image viewed by participants during the test phase.

In humans, a recent fMRI study has provided evidence for specialized neural coding of orientation information based on visual landmarks (Baumann and Mattingley, 2010). Participants in this study undertook an active navigation task within a virtual maze that consisted of multiple parallel corridors intersecting at right angles to form a matrix-like configuration (Figure 3A). Distinctive abstract symbols were located at the end of each corridor. Due to the configuration of the maze, the location of the symbols belonged to one of four possible directions (i.e., North, South, East, West). During learning, participants were trained to navigate accurately and efficiently to all the objects within the virtual maze. In a subsequent test phase, a repetition suppression paradigm was used to compare neural activity across pairs of static images that contained objects from the maze from either the same heading direction (e.g., North–North) or from different directions (e.g., East–North; see Figure 3B). Neural activity in the medial parietal cortex was significantly reduced for objects that corresponded to the same heading direction relative to objects that corresponded to different directions, indicating neural adaptation as a function of heading within this brain area. Whether this activity corresponds directly with head-direction cells in the rodent is uncertain at present. Nevertheless, these findings suggest that the medial parietal cortex is involved in retrieving orientation information elicited by visual landmarks.

The findings reviewed here suggest that environmental objects can provide orientation information for navigation. The processing of directional information involves specialized cells within retrosplenial cortex, as well as in regions of the anterodorsal thalamic nucleus, lateral mammillary nuclei, and the pre- and post-subiculum.

## Objects Used as Associative Cues

The distinction between objects serving as beacons and those that act as associative cues is subtle but important. Whereas the former require only recognition for effective goal localization, the latter must be encoded in such a manner that the object is associated with a relevant navigational context, behavior, or action in order to reach a goal (for a detailed discussion, see Waller and Lippa, 2007). When traveling along an unfamiliar route, for example, objects in the environment might act as cues to prompt an appropriate navigational action (e.g., turn right at the post-office, keep going straight past the church). In support of this view, our ability to learn a novel route between two locations is significantly better for routes that contain salient objects that act as landmarks than for routes without such objects (Heft, 1979; Jansen-Osmann and Fuchs, 2006; Waller and Lippa, 2007). A study of virtual route-navigation by Mallot and Gillner (2000) demonstrated how objects in the environment attain action-related associations. Participants in the task learned to travel a route within a virtual town that contained multiple intersections or decision points, each marked by multiple distinctive landmark objects (buildings). During a subsequent test phase, participants were transported to an intermediate location along the route and were asked to resume the route toward the goal destination. Unbeknownst to participants, the arrangements of buildings along the route were sometimes interchanged within and between different intersections. Notably, participants’ performance was as good as in the trained condition if the new combination of buildings at an intersection was associated with the same directional movement during training (e.g., turn left). Conversely, performance was poorer if the new combination of buildings at an intersection contained buildings associated with a direction that conflicted with that established during training. These findings indicate that environmental objects can become associated with and influence navigational behavior, independent of their actual spatial locations. In contrast to beacon cues, these cues are not a predictor of a single goal location, but provide route information via stimulus-response learning instead. However, like beacon cues, these cues are also thought to be processed by neural networks located in the dorsal striatum (Packard et al., 1989; Packard and McGaugh, 1996).

There is also evidence to suggest that environmental objects can carry additional types of associative information for guiding navigational behavior, which are thought to be stored in the parahippocampal cortex. As discussed above, objects at decision points along a path elicit increased parahippocampal activity relative to objects at non-decision points (Janzen and van Turennout, 2004). This increased activity might reflect the fact that objects at decision points are more likely to be associated with a greater amount of navigation-related information than non-decision point objects. Indeed, other studies have shown that activity in the parahippocampal cortex can be modulated by the number of contextual associations that are elicited by a visual scene (Duzel et al., 2003; Aminoff et al., 2007; Bar et al., 2008). It may be that viewing decision point objects automatically elicits associated information for guiding navigational behavior, which in turn are linked to increased parahippocampal activity.

Overall, the evidence reviewed in this section suggests that environmental objects can be used as associative cues for navigationally related actions. Evidence from human lesion and imaging data, as well as rodent studies, suggest that the use of environmental objects for retrieving associated spatial or navigation-related information appears to rely upon the dorsal striatum and the parahippocampal gyrus.

## Objects Used as a Reference Frame for Navigation

Although the term “landmark” commonly refers to a discrete object within the environment, the geometry of an extended surface or boundary can also provide important information for navigation. In the natural environment, this can refer to the contours of a mountain range or the shoreline, whereas in man-made environments it might refer to the structure of a room or the sides of a large building. In the following section, we provide evidence to show that the geometry of extended surfaces and boundaries, as well as the intrinsic geometry of discrete object locations, can function as a unique landmark by providing a reference frame or schema for the encoding of spatial information. In addition, object geometry can provide orientation and directional information for localizing oneself within a given environment.

Knowledge about the organization of spatial information acquired during navigation has come mainly from findings using object location spatial memory tasks in humans. Early studies argued that object location information was stored in memory like a visual snapshot from a purely egocentric viewpoint (i.e., with reference to the self). Support for this position came from studies showing that spatial recognition performance for remembered object-arrays decreased in a linear fashion as the angular disparity between the presented view and the studied view increased (Diwadkar and McNamara, 1997; King et al., 2002). More recently, it has been shown that although the learned egocentric viewpoint is important, the representation of spatial information can also be organized around environmental reference frames when these are explicitly available (Shelton and McNamara, 2001; Mou et al., 2004). That is, the geometric properties of a large object or building can provide a schema for organizing relevant surrounding object location information. Evidence for this notion has come primarily from studies showing that spatial judgments for a learned object-array is significantly better when made from an imagined orientation that is *aligned* with a salient frame of reference than when made from orientations that are *misaligned* (the “alignment effect,” e.g., Shelton and McNamara, 2001; Mou and McNamara, 2002; Valiquette et al., 2003; Valiquette and McNamara, 2007). Superior performance for aligned versus misaligned orientations is thought to reflect the fact that inter-object spatial relationships are represented in memory with respect to specified reference directions. It has been demonstrated that the geometry of prominent objects like a floor-mat (e.g., Shelton and McNamara, 2001; Valiquette and McNamara, 2007), an enclosed room (e.g., Kelly and McNamara, 2008), a large building (McNamara et al., 2003), or even the intrinsic geometry of discrete object locations (Mou et al., 2008) can provide such frames of reference for encoding object location information.

Given the importance of environmental geometry for establishing frames of reference, it is an open question whether there is a dedicated system for the processing of object geometry. Supporting evidence for the existence of such a dedicated module stems from experiments in rodents. In a seminal paper, Cheng (1986) described a series of experiments in which rats were placed within a rectangular arena with food hidden in one corner. After training, rats were removed from the arena and their established sense of direction was disrupted using a rotation procedure. On probe trials, rats were placed back into the rectangular arena to search for the food that had now been removed. Results consistently showed that the animals searched in the correct corner, but also searched equally often in the corner positioned 180° from the correct (geometrically equivalent) corner. These errors were referred to as “rotational errors,” because the geometrically equivalent location would have been correct if the arena were rotated. Most interestingly, the rats continued to make rotational errors even when unique featural cues, which disambiguated the four corners, were added to the arena. Based on these observations, it was concluded that rats preferentially use geometric cues over featural cues for reorientation. Since the pioneering study by Cheng (1986), many similar findings have been reported in other animal species, including ants, fishes, birds, primates, and humans (for a review, see Cheng and Newcombe, 2005). Young children, for example, display similar rotational errors after disorientation when searching for a toy within a rectangular room, even when featural cues are available to distinguish the two corners (Hermer and Spelke, 1994). These findings have led to the proposal that there may be a dedicated system – a “geometric module” – in both humans and non-human animals, that automatically encodes geometric boundary information for orientation and navigation (Cheng, 1986; Wang and Spelke, 2002; Lee and Spelke, 2010). It is argued that the processing of boundary information represents a distinctive system that is separate from that which subserves the encoding of discrete landmarks (Wang and Spelke, 2002). Although adults are able to use both geometric and non-geometric information for goal localization, there is evidence that the ability to point to a configuration of local landmarks is significantly impaired after disorientation, whereas their ability to point to the corners of a room is intact (Wang and Spelke, 2000). It is important to note that these studies do not imply that children and animals cannot utilize featural cues. Instead, they demonstrate that under certain conditions children and animals can exhibit behaviors suggesting that they prefer to use the geometric properties of a surrounding boundary.

Further evidence for the existence of a specialized system for the encoding of geometric environmental information comes from human fMRI investigations. In two parallel studies, Doeller and colleagues showed that spatial learning based upon boundary information and discrete proximal landmarks involve two distinct sets of behavioral and neural processes (Doeller and Burgess, 2008; Doeller et al., 2008). In both studies, participants had to learn the locations of target objects in a virtual environment, using either the surrounding circular boundary or a discrete landmark placed near the target. Between blocks of trials, half the target objects maintained their positions relative to the landmarks, and the other half of the objects maintained their positions with respect to the circular boundary. Learning trials for boundary-related target objects were associated with increased hippocampal activation, whereas trials concerning discrete proximal objects were associated with increased striatal activity (Doeller et al., 2008). Furthermore, in a purely behavioral version of the task, participants learned to use the navigational information provided by the boundary incidentally, even in task manipulations in which encoding of boundary information was neither encouraged nor required (Doeller and Burgess, 2008). In a recent fMRI study, having participants simply imagining empty visual scenes with a discrete, “wall-like” object (i.e., an elongated block) resulted in an increase in hippocampal activity compared with imagining a scene without the object (Bird et al., 2010). Increasing the number of wall-like objects in the imagined scene resulted in an increase in the amount of hippocampal activity, again supporting the notion that hippocampal activity reflects, at least to some extent, the amount of boundary information present in an environment. Together, these findings suggest that geometric information may be encoded automatically by the hippocampus during navigation.

These findings in humans are further corroborated by neurophysiological rodent research. An important study by O’Keefe and Burgess (1996) showed that changes to the geometric properties of an environment might result in relative changes in place cell firing. Expansion of rectangular environments resulted in parametric stretching and reforming of place fields. This initial discovery led to a model of spatial processing that postulates that place cell firing in the hippocampus reflects the outcome of accumulated activity of multiple cells, termed “boundary vector cells,” which are tuned to the relative distance and direction of environmental boundaries (Burgess et al., 2000; Barry et al., 2006). In support of this computational model, cells with similar response characteristics as predicted by the model have been described in the subiculum and medial entorhinal cortex of the rodent (Solstad et al., 2008; Lever et al., 2009). It has also been shown that the shape of environmental boundaries modulates the neuronal firing patterns in these brain regions (Lever et al., 2009), indicating that physical properties of environmental geometry are important factors to be considered when investigating navigation behavior.

However, it is important to note that hippocampal involvement in spatial navigation processing is not unique to geometric information. In humans as well as rodents it has been shown that the processing of inter-object relationships between discrete distal objects has also been shown to depend upon the hippocampus (Save and Poucet, 2000; D’Hooge and De Deyn, 2001; Thomas et al., 2001; Astur et al., 2002; Oswald et al., 2003; Parslow et al., 2004).

In conclusion, environmental geometry possesses unique properties that are useful for providing a spatial reference frame for navigation. As highlighted, extended surfaces and boundaries in particular can influence the organization of spatial information in memory and can have a significant impact on place cell representations. The use of global environmental reference frames can also be considered as a logical prerequisite for the formation and storage of survey representations or so-called cognitive maps, which are also thought to be hippocampal-dependent cognitive processes (O’Keefe and Nadel, 1978; Maguire et al., 1999; Bird and Burgess, 2008).

## Proposed Taxonomy of Landmark Functions

Based on the research findings outlined above, we propose that objects encountered during navigation can be grouped into four distinct classes of landmarks based on their behavioral function and related neural correlates (Table 1). At its simplest, an environmental object can act as a beacon, marking an exact or nearby goal location. Beacon-based navigation appears to involve simplistic allocentric mechanisms supported by the striatum. Objects can also provide information about one’s current heading orientation. In rodents, specialized cells have been found in the retrosplenial cortex, as well as in regions of the anterodorsal thalamic nucleus, lateral mammillary nuclei, pre- and post-subiculum, that respond to the animal’s heading based on available visual cues. Orientation-based neural activity has also been found in the human medial parietal cortex (Baumann and Mattingley, 2010). At a more semantic level, environmental objects can be used as associative cues for eliciting navigationally relevant contextual information. For example, objects can be associated with a specific navigational action learned through experience (e.g., turn right), or provide other relevant information supporting successful navigational behavior. These associative processes appear to rely on the striatum (Packard et al., 1989; Packard and McGaugh, 1996) as well as the parahippocampal cortex (e.g., Janzen and van Turennout, 2004; Bar et al., 2008). Finally, the geometry of visual environmental boundaries and object locations can act as landmarks, by providing a reference frame for the encoding of object location information. Similar to the storage of survey representations or so-called cognitive maps, the encoding of environmental geometry is related to hippocampal activity.

**Table 1:** **Proposed four-part taxonomy of landmark function in navigation**.

Landmark type	Neural correlate	Functional description
Beacon	Striatum	Single object that marks the exact or nearby location of a goal
Orientation cue	Retrosplenial cortex, anterodorsal thalamic nucleus, and pre- and post-subiculum	Visual cue that provides information about one’s current heading direction
Associative cue	Striatum and parahippocampal gyrus	Single object that is associated with navigationally relevant information
Reference frame	Hippocampus	Environmental geometry (extended surfaces, boundaries, and the intrinsic geometry of discrete object locations) that provides a framework for spatial encoding and localization

## Conclusion

Visual information plays an important role in guiding navigation behaviors. In this review, we have proposed a four-part nomenclature for categorizing the use of objects as landmarks for navigation. Each landmark type has been defined based on behavioral evidence and related neural correlates. By focusing on object function rather than form, we have emphasized that any environmental object can serve as a landmark. The particular navigational function of an object or cue will depend upon the environmental context and the goals and preferences of the individual (Steck and Mallot, 2000). Individual differences in navigational abilities can also influence the type of landmark information that is encoded (Wolbers and Hegarty, 2010).

We hope the taxonomy outlined in this review will provide a useful point of reference for future research on landmark-based spatial navigation. The schema we have described should serve as a useful guide for investigators who wish to define and quantify the functional role of visual objects during navigation in real-world and laboratory based studies. Providing a clear terminology for categorizing landmark function may also be useful in the clinical domain, in which acquired deficits in navigation behavior are often encountered (e.g., in stroke and Alzheimer’s disease; e.g., Barrash et al., 2000; Gazova et al., 2012). A clearer conception of the functional role of visual landmarks might even inspire the development of novel intervention strategies for individuals with navigational impairments (e.g., Caglio et al., 2012).

## Conflict of Interest Statement

The authors declare that the research was conducted in the absence of any commercial or financial relationships that could be construed as a potential conflict of interest.
